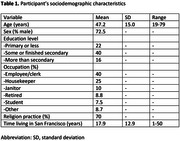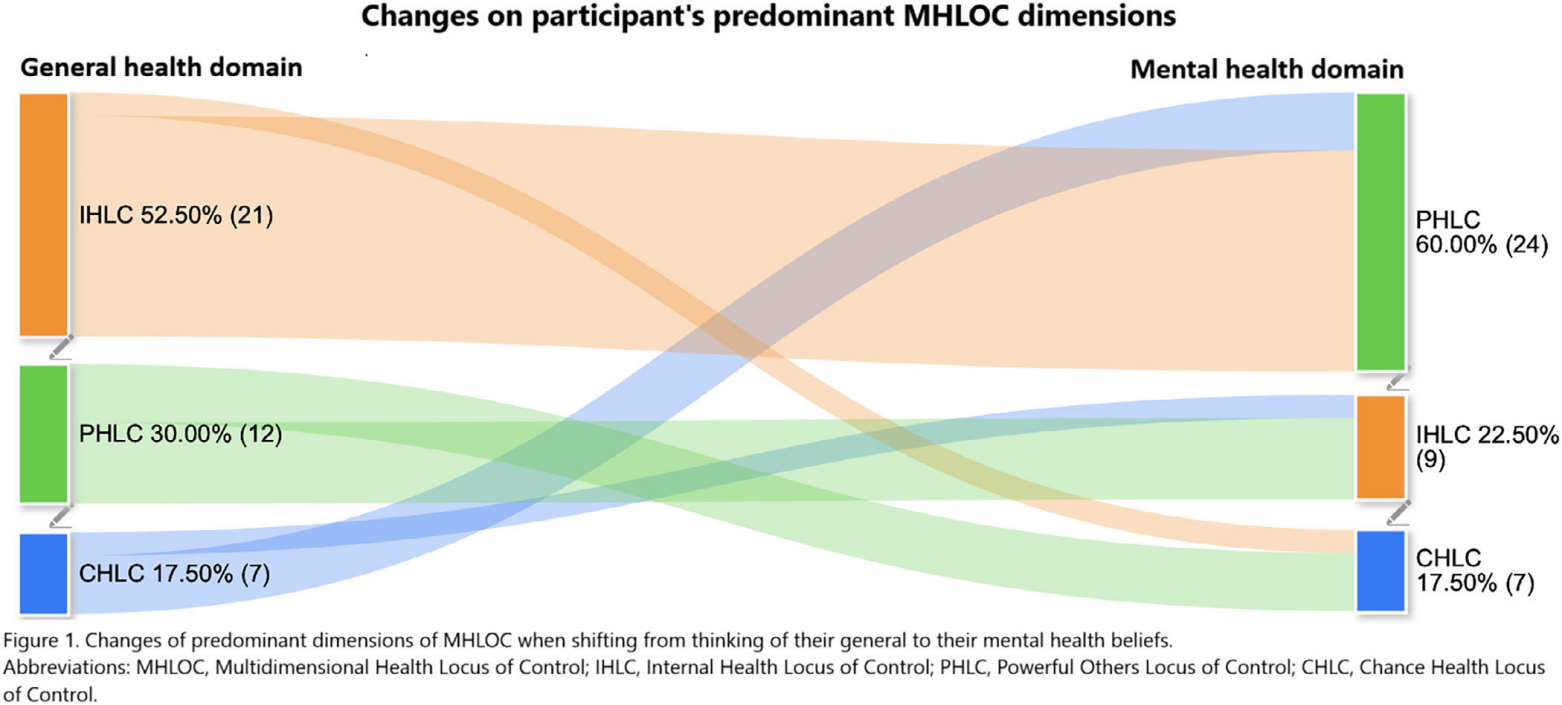# General Health and Mental Health Locus of Control in a Diverse Sample of Latino San Franciscans Attending a Community‐Based Primary Care Clinic

**DOI:** 10.1002/alz70860_107480

**Published:** 2025-12-23

**Authors:** Sonia Leonor Sanchez Mendoza, Luis E Martinez, Gloria A. Aguirre, Berenice Fuentes‐Juarez, Isabel Elaine Allen, Serggio Lanata

**Affiliations:** ^1^ Global Brain Health Institute, University of California, San Francisco, CA, USA, San Francisco, CA, USA; ^2^ Memory and Aging Center, UCSF Weill Institute for Neurosciences, University of California, San Francisco, San Francisco, CA, USA; ^3^ Global Brain Health Institute, University of California, San Francisco, CA, USA, San Francisco, CA, USA, San Francisco, CA, USA; ^4^ Memory and Aging Center, UCSF Weill Institute forNeurosciences, University of California, San Francisco, San Francisco, CA, USA, San Francisco, CA, USA; ^5^ Department of Epidemiology and Biostatistics, University of California, San Francisco, San Francisco, CA, USA; ^6^ Global Brain Health Institute, University of California, San Francisco, San Francisco, CA, USA

## Abstract

**Background:**

A more integrated approach to mental and brain health across the lifespan is needed. Understanding how individuals in a specific community perceive control over their health outcomes may provide insights for multidimensional and personalized care. We used the Multidimensional Health Locus of Control scale (MHLOC) to assess a group of Spanish‐speaking, Latino San Franciscans. Beliefs on what influences their general and mental health were measured in three dimensions: Internal Health Locus of Control (IHLOC), Powerful Others Health Locus of Control (PHLOC), and Chance Health Locus of Control (CHLOC).

**Method:**

Participants provided informed consent and self‐administered the MHLOC scale in the San Francisco (SF) Mission Neighborhood Health Clinic. Sociodemographic variables were collected (age, sex, education, years living in SF, religion). We compared participants’ general and mental health beliefs and tested for associations between their MHLC dominant dimension and their sociodemographic variables.

**Result:**

78 participants (47.2 years, 72.5% male) with diverse sociodemographic backgrounds completed the MHLOC scale (table 1). PHLOC dimension was dominant for general and mental health (43% and 51%, respectively), followed by IHLOC (31% and 33%), and CHLC (26% and 17%). A large proportion of participants changed their dominant dimension when shifting from thinking of their general to their mental health beliefs (*n* = 40, 51.3%). The most prominent change was from IHLC to PHLC dominance (*p* = 0.03, Figure 1). Age and education were found to have a significant effect on general health beliefs (one‐way ANOVA, F = 4.50, *p* = 0.014 and *χ* ^2^ = 26.35, *p* = 0.010, respectively).

**Conclusion:**

Our findings suggest a tendency among Latino San Franciscans to view their mental health as strongly influenced by external factors, while viewing their general health as strongly influenced by self‐responsibility. Acknowledging and supporting participants’ reliance on external resources while fostering self‐efficacy could guide prevention and intervention strategies for mental and brain health.